# A Rare Case of a Non-seminomatous Testicular Cancer Metastasis to the Duodenum: A Case Report

**DOI:** 10.7759/cureus.59332

**Published:** 2024-04-30

**Authors:** Anas K Assi, Habeeb H Awwad, Nora I Baraghithi, Zaina A Khaled, Abdulrahim M Odeh

**Affiliations:** 1 Faculty of Medicine, Al-Quds University, Jerusalem, PSE; 2 Gastroenterology and Hepatology, Istishari Arab hospital, Ramallah, PSE

**Keywords:** lymph nodes, vomiting, epigastric pain, duodenum metastasis, radical orchiectomy, embryonal testicular cancer

## Abstract

Testicular cancer is among the most common solid tumors in young men. Gastrointestinal tract (GIT) metastasis of testicular cancer has been rarely reported. In addition, metastasis occurs most commonly through retroperitoneal lymph nodes. Manifestations like abdominal pain and obstruction can be present if metastasis to GIT was considered. We report here a case of a 34-year-old male who was admitted to our GIT unit complaining of episodic epigastric pain. Computed Tomogram (CT) scan demonstrated a soft tissue like lesion involving the lumen of duodenum. Moreover, the patient had a right radical orchiectomy 18 months prior to the presentation due to a stage IA non-seminomatous germ cell tumor with no lymphovascular invasion and free surgical margins. Esophagogastroduodenoscopy (EGD) revealed a malignant appearing duodenal lesion and biopsy showed that it was compatible with germ cell tumor. Metastatic embryonal carcinoma to duodenum was diagnosed and confirmed by immunohistochemical stains. Then, the patient’s situation was discussed and decided to be on a plan of four cycles of chemotherapy regimens. Testicular malignancy metastasis to GIT is uncommon, but it’s important to know that there is a contact between GIT and testicular lymphatic drainage through para-aortic lymph nodes. So, even if it’s rare to occur, it’s still possible, and we should always be concerned about it. Mostly, diagnosis of testicular tumors begins with evaluating tumor markers such as alpha-fetoprotein (AFP), beta-subunit of human chorionic gonadotropin (B-hCG), and lactate dehydrogenase (LDH). But in contrast, all of these markers were within the normal range of their values in our case. Suspicion for metastasis and GIT involvement must be raised when dealing with a young male who had a history of testicular tumor such as embryonal carcinoma which was reported here in our case. That is very essential for avoiding potential complications and saving time in order to start management.

## Introduction

One of the most common solid tumors among young men is testicular cancer [1]. Seminomas and non-seminomas are two types of germ cell cancers. Seminomas are substantially less likely than non-seminomatous germ cell cancers to spread into the gastrointestinal tract (GIT). Nevertheless, it's crucial to understand that cases of testicular cancer spreading to the GIT are extremely rare [2]. When it comes to testicular cancer metastasis, hematogenous and lymphatic drainage channels are typically common pathways [2]. Direct extension from the retroperitoneal lymph nodes, which drain the testis, is the most common mechanism of metastasis [1]. Generally, adenocarcinoma is the main primary cancer of the duodenum, and metastasis from other tumors accounts only for 16.3% of duodenal tumors [3]. We report here a case of a 34-year-old man with a history of right testicular tumor, who later on started to complain of abdominal pain. After that, a duodenal mass was discovered and was also seen on a CT scan. A biopsy verified metastasis to the duodenum, which was consistent with a non-seminomatous germ cell tumor on histopathology.

## Case presentation

A 34-year-old Palestinian male patient works in a garage and is a 20-pack/year smoker. The patient was referred to our GIT unit in February 2024 complaining of episodic recurrent epigastric pain for three months, the pain was throbbing in nature, gradual onset, and radiated to the right side of his abdomen. It was associated with coffee ground non-projectile vomiting, about four cups in amount. The pain was exacerbated by sitting forward and relieved by standing and walking. Additionally, he reported significant weight loss, nausea, and a decrease in appetite. He denied any other GIT symptoms.

Upon admission, physical assessment was done and the patient looked reasonable, afebrile with normal vital signs (Temperature 36.8 degrees, O_2_ sat 98% on room air, blood pressure 135/82 supine, right arm), otherwise unremarkable except for a right sided surgically removed testicle.

**Figure 1:**
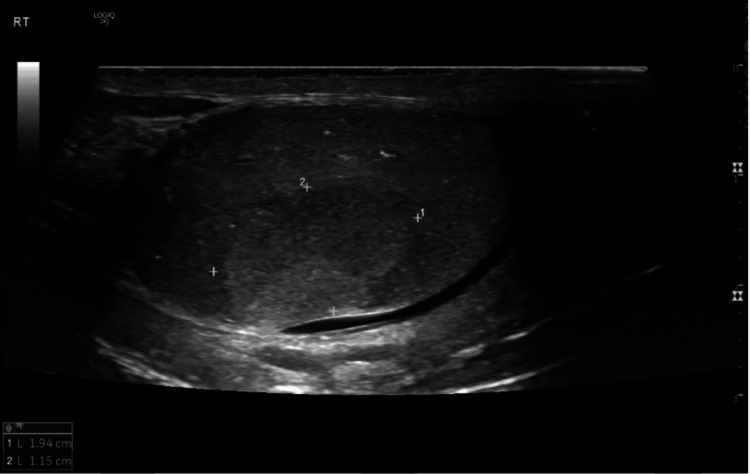
A right scrotal Doppler ultrasound U/S showed a well-defined vascularized heterogeneous mass measuring 2×1.2 cm in size, and there were multiple variable-sized hyperechoic areas with posterior shadowing.

His surgical history started in September 2022 when the patient was referred from an outpatient clinic to Palestine Medical Complex hospital for consultation about an abnormal testicular mass. One year prior to his referral, he had noticed a small, non-tender mass in his right testicle; the mass didn’t enlarge before seeking any consultation. He didn’t have any fever, constitutional symptoms, or family history at that time. A scrotal Doppler ultrasound was performed (Figure 1). It showed a well-defined vascularized heterogeneous mass measuring 2 × 1.2 cm in size, and there were multiple variable-sized hyperechoic areas with posterior shadowing. The left testicle was normal. A CT scan was performed, and the result was unremarkable except for the previously mentioned mass.

A right radical orchiectomy through a subinguinal incision was done in September 2022 after a full biological evaluation, including negative tumor markers and a free thoraco-abdominal-pelvic CT. The patient consents to preserve semen samples in the semen bank.

**Figure 2:**
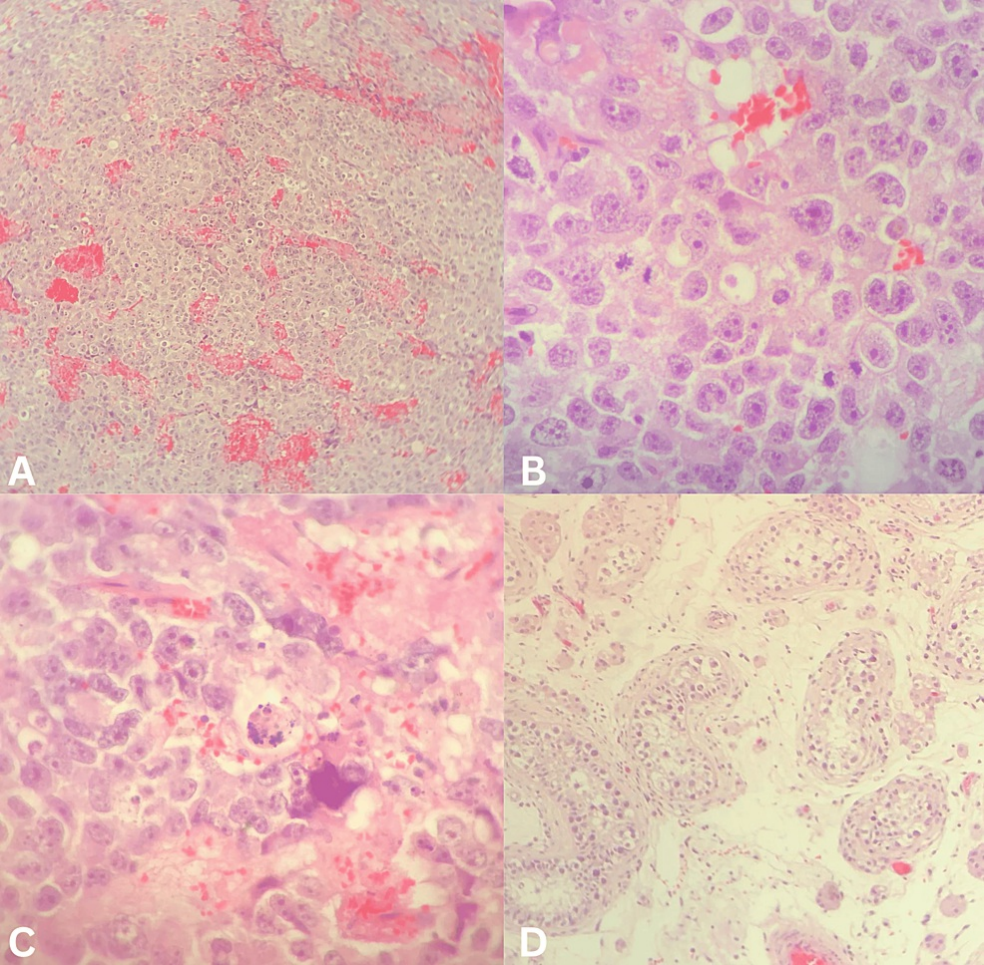
An anatomopathological examination An anatomopathological examination revealed a tumor composed of cohesive clusters of anaplastic epithelial cells with a solid and tubular pattern. These cells have abundant cytoplasm and large vesicular pleomorphic nuclei with prominent macronuclei. (a) Foci of coagulative necrosis and hemorrhage (H&E, 100x); (b) a high mitotic rate (H&E, 400x); (c) and karyorrhectic fragments are seen (H&E, 400x); (d) there was no invasion of the epididymis or the spermatic cord, and the non-neoplastic seminiferous tubules showed atrophic changes with no spermatogenesis (H&E, 100x). H&E: hematoxylin and eosin

The histopathological findings of the resected testis showed a pure embryonal carcinoma limited to the testis with no lymphovascular invasion and free surgical margins, there was no invasion of the epididymis or the spermatic cord, and non-neoplastic seminiferous tubules showed atrophic changes with no spermatogenesis (Figure 2). Further immunohistochemical staining tests were requested and showed tumor cells that are diffusely positive for CD30 and OCT3/4, focally positive for cytokeratin7 (CK7) and placental alkaline phosphatase (PLAP), and negative for epithelial membrane antigen (EMA) immunostaining which is also consistent with embryonal carcinoma.

The patient’s final clinical diagnosis was stage IA non-seminomatous germ cell testicular cancer (NSGCT. IA) so he was an acceptable candidate for active surveillance only which included serial physical examinations, laboratory tests, and radiological studies every three months.

**Figure 3:**
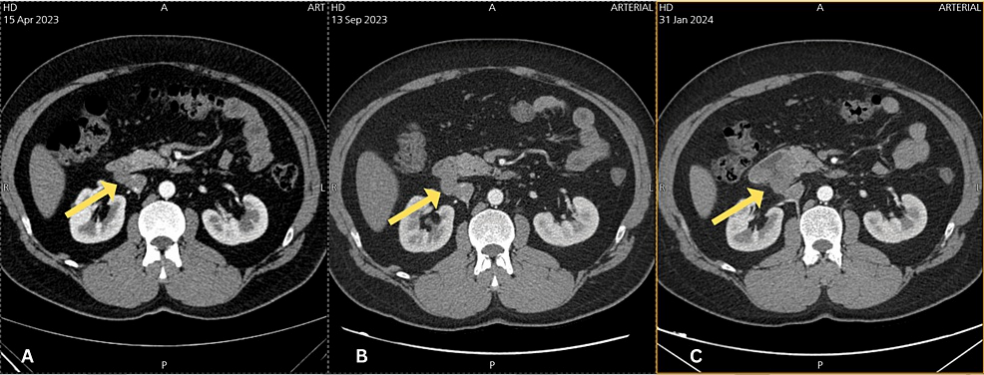
Coronal view of multiple CT scans with IV contrast showing the progressively enlarging mass (yellow arrows) (a) In April 2023, a single enlarged paracaval lymph node was seen and measured about 12 mm in maximum short axis. (b) In September 2023, the lymph node had showed an 18% increase in its size and measured about 2×1.6 cm. (c) In January 2024, a newly appearing soft tissue like lesion involved the lumen of the second part of the duodenum and it was inseparable from the paracaval lymph node.

In April 2023, a CT scan with intravenous contrast was performed and a single enlarged paracaval lymph node was seen and measured about 12 mm in maximum short axis. In September 2023, the lymph node had showed an 18% increase in its size and measured about 2×1.6 cm. In January 2024, an abdominal and pelvic CT scan with IV contrast was repeated and compared with previous CT scan that was done in September 2023, the results showed a newly appearing soft tissue like lesion involving the lumen of the second part of the duodenum and the paracaval lymph node could not be separated from the above mentioned new process, so it could be a non-separable lymph node or an exophytic part of duodenal lesion (Figure 3). Consequently, an endoscopy was demanded for duodenal biopsy taking. 

Before endoscopy in February 2024, all basic laboratory investigations were conducted for the patient. His HGB was 14.4 g/dl, his hematocrit (HCT) was 43.4%, mean corpuscular volume (MCV): 90.4 fl, platelets count 402 K/μl, creatinine 0.93 mg/dl, blood urea nitrogen (BUN): 15, thyroid function tests were within the normal range (thyroid-stimulating hormone (TSH): 1.516 mTu/ml), there was a mild elevation in the uric acid 7.5 mg/dl) and C-reactive protein 11.6, while the alpha-fetoprotein (AFP), carcinoembryonic antigen, CA 15-3, CA 19-9 and prostate specific antigen were all normal.

**Figure 4:**
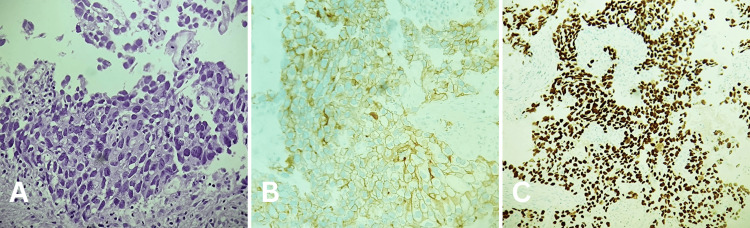
A biopsy from the second part of the duodenum with immunohistochemical stains The duodenum is infiltrated by solid sheets of polygonal crowded cells that have indistinct and distinct cell borders with overlapping nuclei and high-grade nuclear features. Numerous mitotic figures are present. Smudgy degenerative appearing nuclei are seen. Necrosis is present (a) (H&E, 100x). The neoplastic cells are positive for CD30 (b) (magnification 100x), SALL4 (c) (magnification 100x) immunostains. H&E: hematoxylin and eosin; SALL4: Sal-like protein 4

Endoscopy revealed a malignant appearing duodenal lesion at the second part of the duodenum occupying half of the lumen, a biopsy sample was taken and showed a malignant germ cell tumor (metastatic embryonal carcinoma). The duodenum was infiltrated by solid sheets of polygonal crowded cells that have indistinct and distinct cell borders with overlapping nuclei and high-grade nuclear features. Numerous mitotic figures and smudgy degenerative appearing nuclei were seen. Necrosis was present and the neoplastic cells are positive for CD30, SALL4 and OCT3/4 immunostains (Figure 4). A plan of four cycles of bleomycin, etoposide, and cisplatin (BEP) chemotherapy regimens were discussed with the patient.

## Discussion

Testicular neoplasms, accounting for only 0.5% to 1% of all solid male cancers, are the most common malignancy among males aged 15 to 35 [4]. Their incidence varies by race, with a higher prevalence in white individuals compared to Africa and Asia [2,5]. Germ cell tumors, the predominant type of testicular tumor, consist of two main histologies: seminoma (SEM) and non-seminomatous testicular tumor (NSE). Embryonal carcinoma, choriocarcinoma, teratoma, and yolk sac tumors are included in NSE. Approximately 15% of germ cell tumors are mixed tumors, containing both seminoma and non-seminoma elements. Seminomas are typically more confined to the testes and usually emerge later in life, with a mean age of 35 years old at first presentation, compared with 25 years of age for NSE [2].

Both genetic and environmental factors increase the risk of testicular cancer, including cryptorchidism, a family history with a six to 10 times higher risk in first-degree relatives, contralateral testicular tumors, hypospadias, and decreased spermatogenesis due to subfertility or infertility [5,6]. Despite this, our patient did not exhibit any of these risk factors.

Testicular masses typically manifest as painless swelling in one testis, often accompanied by dull abdominal or scrotal pain. About 5% of patients may develop gynecomastia, likely due to elevated human chorionic gonadotropin (HCG) levels. While 10% may experience acute testicular pain. Additionally, symptoms of metastasis can also be present at the initial presentation. NSE tends to spread lymphatically, except for choriocarcinoma, which metastasizes hematogenously [5]. Embryonal carcinoma has the potential to disseminate via lymphatic and hematologic routes, with the most frequent lymph node metastases occurring in the lumbo-aortic region. Heterogeneous metastatic dissemination predominantly involves the lungs, followed by the liver, and less frequently, the brain and bones. However, gastrointestinal metastasis is more common in non-seminomatous tumors, occurring through direct spread into the retroperitoneal lymph nodes [2,4]. Therefore, the metastasis to the second part of the duodenum which is a rare site is what makes our case special.

Diagnosis of testicular tumors begins with assessing tumor markers such as AFP, B-hCG, and lactate dehydrogenase (LDH). Embryonal carcinoma is capable of producing both AFP and HCG [5,7]. Unlike the case report, the laboratory tests illustrate a normal range of tumor markers. Trans-scrotal ultrasound is the preferred initial imaging modality; a hypoechoic, solid, vascularized intratesticular lesion is typically indicative of testicular cancer. A definitive diagnosis requires a radical inguinal orchiectomy to perform a histopathological examination and immunohistochemical staining. Embryonal carcinoma staining is positive for OCT 3/4, CD30, and SALL4 [8,9]. Additionally, all patients should undergo contrast-enhanced CT of the abdomen and pelvis to rule out possible metastasis [4].

The staging of testicular tumors is based on the American Joint Committee on Cancer (AJCC) group. Stage IA tumors are described as limited neoplastic cells in the testicles without lymphovascular invasion and normal tumor marker levels [10]. Radical inguinal orchiectomy is curative in 75% of cases. Furthermore, active surveillance, which involves serial examinations, laboratory tests, and imaging studies, is required due to the risk of relapse in 35% of patients. Although 65% overall will not demonstrate any metastasis or relapsing over the next 5 years, the overall 5-year survival rate after excision and chemotherapy is about 92% [5].

Micrometastasis may be present at the time of orchiectomy, increasing the risk of testicular tumor relapse. The risk for micrometastasis is higher with both embryonal carcinoma and a high level of AFP [5]. Gastrointestinal metastasis of testicular tumors is a rare occurrence, and it may present with variable clinical features. Our patient complained of epigastric pain along with nausea and vomiting. Consequently, an upper gastrointestinal endoscopy and biopsy were performed, revealing metastatic embryonal testicular carcinoma. Therefore, the possibility of duodenal metastasis should be considered before hazardous complications occur. Metastatic testicular tumors have shown a response to chemotherapy regimens such as BEP chemotherapy [5].

## Conclusions

In summary, the duodenum is a rare site for non-seminomatous testicular cancer metastases. When a patient has a history of testicular tumor and presents with gastrointestinal symptoms such as abdominal pain, nausea, and vomiting, it is important to be suspicious of metastases. Prompt detection and management are essential to prevent all potential complications. This case contributes to the literature by highlighting the possibility of metastasis to a rare site.
